# Partial response to erlotinib in a patient with imatinib-refractory sacral chordoma

**DOI:** 10.1186/s13569-020-00149-1

**Published:** 2020-12-12

**Authors:** Saurav Verma, Surya Prakash Vadlamani, Shamim Ahmed Shamim, Adarsh Barwad, Sameer Rastogi, S. T. Arun Raj

**Affiliations:** 1 Department of Medical Oncology, Institute Rotary Cancer Hospital, All India Institute of Medical Sciences, New Delhi, India; 2 Department of Nuclear Medicine, All India Institute of Medical Sciences, New Delhi, India; 3 Department of Pathology, All India Institute of Medical Sciences, New Delhi, India; 4 Sarcoma Medical Oncology Clinic, All India Institute of Medical Sciences, New Delhi, India

**Keywords:** Chordoma, EGFR, Erlotinib

## Abstract

**Background:**

Chordoma is a rare, slow growing and locally aggressive mesenchymal neoplasm with uncommon distant metastases. It is a chemo-resistant disease with surgery and radiotherapy being the mainstay in treatment of localized disease. In advanced disease imatinib has a role. We report a case of metastatic sacral chordoma with symptomatic and radiological response to erlotinib post-progression on imatinib.

**Case presentation:**

A 48-year-old male with a sacral chordoma underwent partial sacrectomy followed by post-operative radiotherapy. Upon recurrence he received palliative radiotherapy to hemipelvis and was offered therapy with imatinib. However, the disease was refractory to imatinib and he was started on treatment with erlotinib—showing a partial response on imaging at two months. He is currently doing well at 13 months since start of erlotinib.

**Conclusions:**

As seen in previously reported cases, erlotinib is a therapeutic option in advanced chordoma, even in imatinib refractory cases and thus warrants exploration of its therapeutic role in prospective clinical trials.

## Background

Chordoma is a rare mesenchymal neoplasm which arises from the remnants of primitive notochord [1]. It accounts for 2–4% of all malignant bone tumors and the median age of presentation is 59 years (range 19–70 years) with a male predominance [2, 3]. They occur exclusively in spine, predominantly at sacrococcygeal and spheno-occipital areas, at a median or paramedian location [4]. It is a slow growing locally aggressive tumor with uncommon distant metastases, athough it can cause compressive symptoms.

Surgery significantly improves overall survival and is the primary modality of therapy for the localized disease [5]. Radiotherapy also plays a key role in the management of patients with localized chordoma, particularly in the adjuvant setting after a full or subtotal resection, and as the primary treatment in unresectable disease.

Chordoma responds poorly to cytotoxic chemotherapy. Azzarelli et al. in a case series of 33 patients, concluded that none of the chemotherapeutic regimen induced a significant tumor response [6]. This intrinsic chemo-resistance of chordoma paved the way to different antitumor approaches.

In patients with advanced disease, novel therapeutic strategies are needed to prolong survival and improve the quality of life. Imatinib, which has off-target effects acting through the inhibition of platelet-derived growth factor receptor beta (PDGFRβ), is the most thoroughly evaluated therapeutic agent in chordoma, based on the expression of platelet-derived growth factor beta (PDGFβ) or its receptor (PDGFRβ) [7, 8].

There is preclinical evidence of the role of epidermal growth factor receptor (EGFR) in chordoma pathogenesis and also a few case reports elucidating the role of blocking this receptor  leading to meaningful clinical responses [9–12]. Herein we present a case of metastatic sacral chordoma that has shown response to erlotinib after having progressed on imatinib.

## Case presentation

**Fig. 1:**
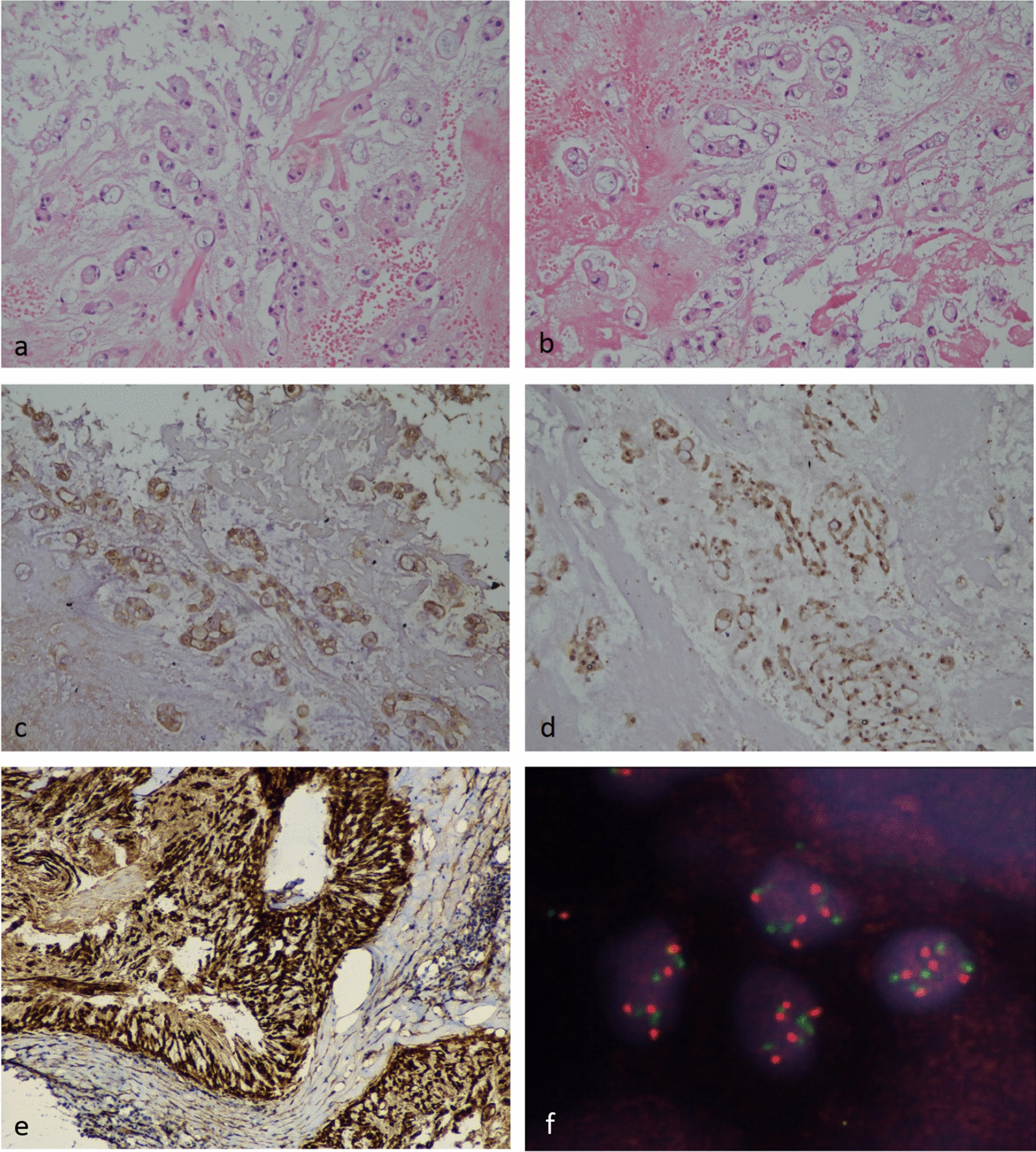
**a**, **b** Histological picture showing a tumor with chondromyxoid background. The tumor cells are arranged in cord like pattern with small nucleus and abundant cytoplasm. Few cells are large with abundant vacuolated cytoplasm called as physaliferous cells. **c** Immunostaining for S100 protein showing nuclear positivity in the tumor cells. **d** Immunostaining for Epithelial Membrane Antigen (EMA) showing membranous positivity in the tumor cells. **e** Immunostaining for epidermal growth factor receptor (EGFR) showing membranous positivity in the tumor cells. **f** MET gene amplification was positive by FISH

**Fig. 2:**
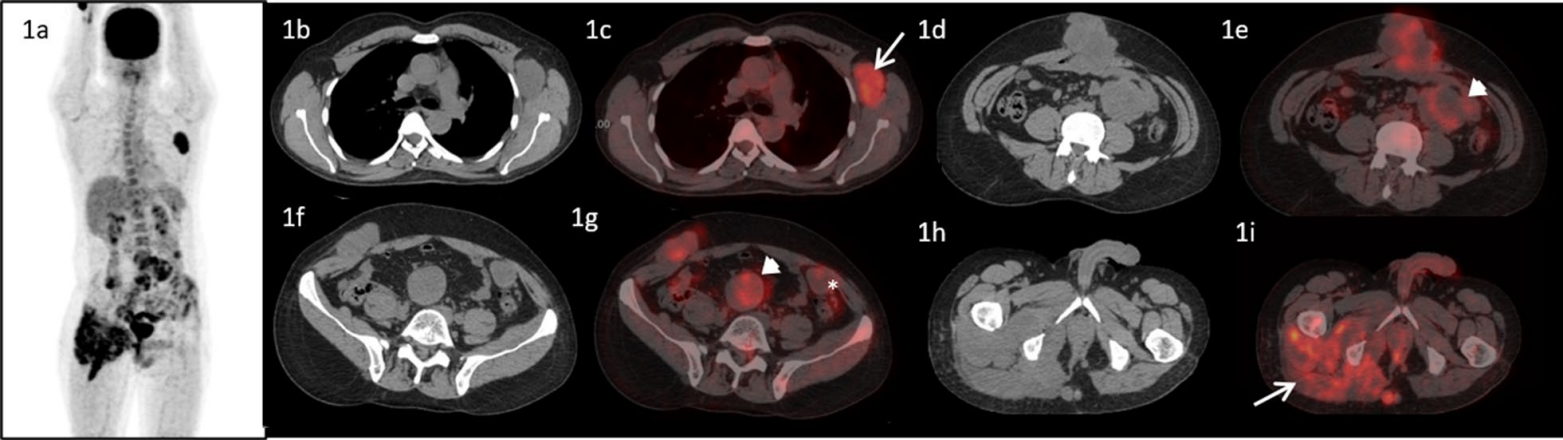
A pre-imatinib 18F-Flurodeoxy glucose (FDG) positron emission tomography/computed tomography (PET/CT) maximum intensity projection (MIP) image of the patient showing increased FDG uptake in the left axillary, lower abdominal and pelvic regions. Transaxial computed tomographic images at the level of axilla, L3 vertebra, L5 vertebra and ischial tuberosity (**1b**, **1d**, **1f** and **1h**) and corresponding fused PET/CT images (**1c**, **1e**, **1g** and **1i**) showing enlarged and FDG avid left axillary lymph node (**1c**, arrow), FDG avid lobulated mass in the anterior abdominal wall (**1d** and **1e**), deposit in the right external oblique muscle (**1f** and **Ig**), multiple mesenteric (arrow heads) and omental* deposits (**1e** and **1g**). There was soft tissue thickening adjacent to the right ischium involving the right pyriformis and gluteal muscles with increased FDG uptake and soft tissue deposit the subcutaneous plane adjacent to it (**1h**, **1i** -arrow)

**Fig. 3.**
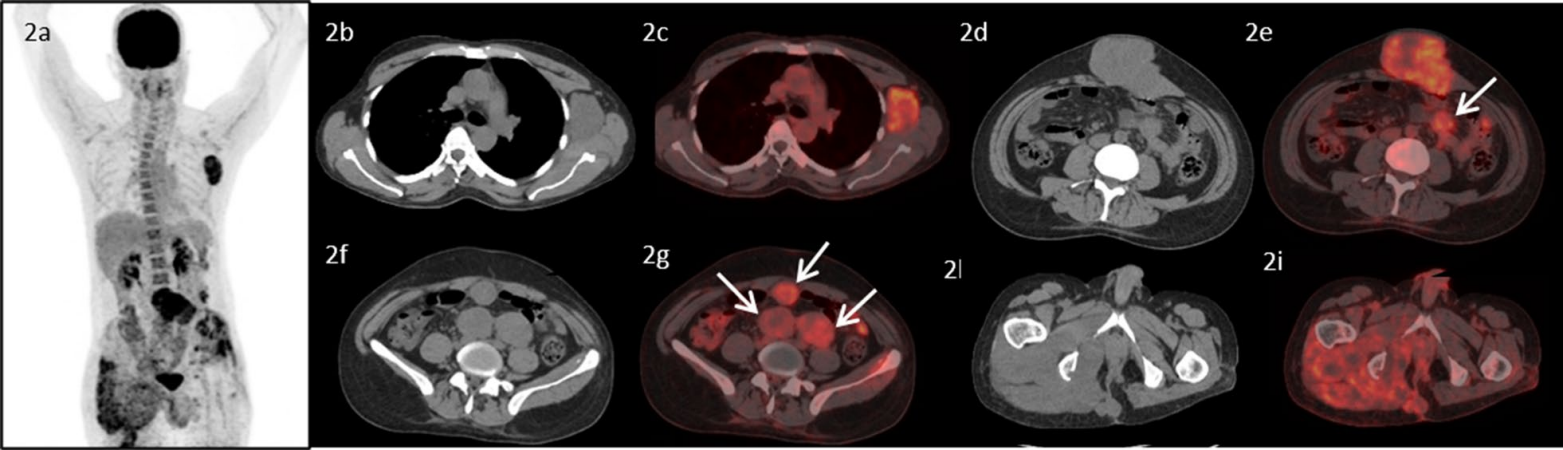
18F-FDG PET/CT images of the patient three months after initiation of therapy with imatinib. MIP image (**2a**) shows focal increased FDG uptake in the left axillary region, lower abdominal and pelvic regions, which increased since the previous PET/CT image (**1a**). Transaxial CT and fused PET/CT images show increase in size and metabolic activity of the of the left axillary lymph node (**2b** and **2c**), anterior abdominal wall lesion (**2d** and **2e**), with interval appearance of new omental/mesenteric deposits (**2e**, **2g**, arrows). No significant interval change was noted in the lesion near the right ischium

**Fig. 4.**
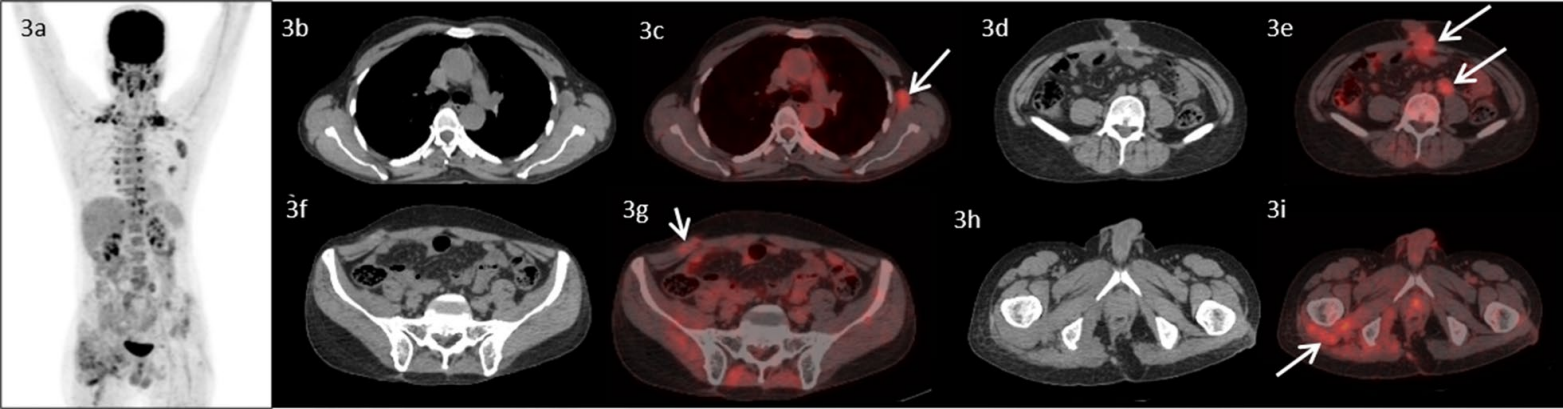
18F-FDG PET/CT images of the patient two months after initiation of therapy with Erlotinib. MIP image (**3a**) shows mild focal FDG uptake in the left axillary region, lower abdominal and pelvic regions, which decreased since the previous PET/CT image (**2a**). Transaxial CT images (**3b**, **3d**, **3f**, **3h**) show significant reduction in size of the left axillary lymph node, anterior abdominal wall lesion and omental/mesenteric deposits metastatic lesions (partial response, RECIST criteria) and fused PET/CT images (**3c**, **3e**, **3f**, **3i**- arrows) show decrease in metabolic activity of the of the corresponding lesions suggestive of partial metabolic response

**Fig. 5.**
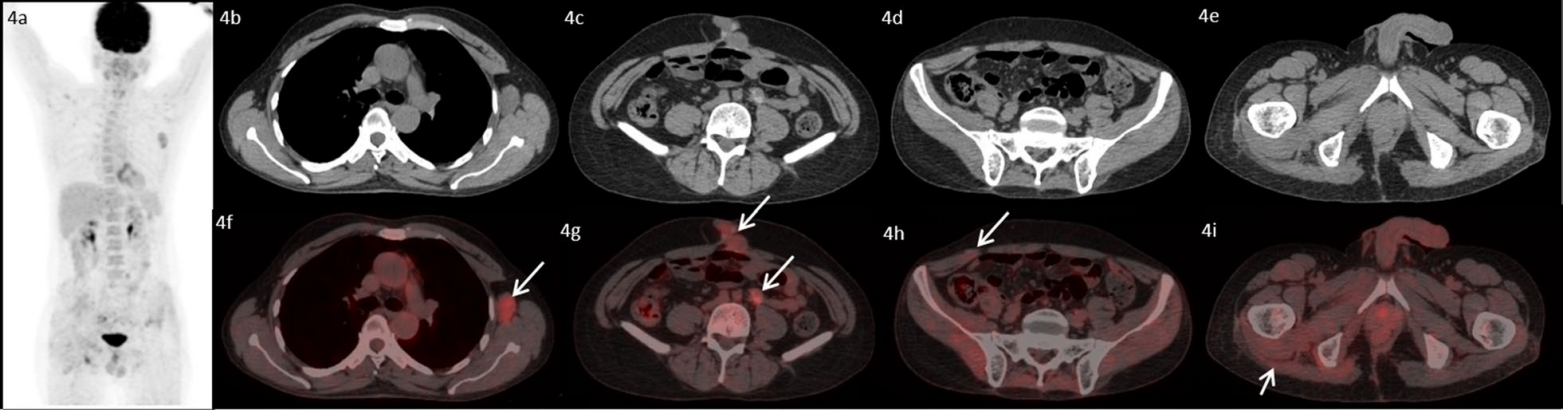
18F-FDG PET/CT images of the patient six months after initiation of therapy with erlotinib. MIP image (**4a**) transaxial CT images (**4b**–**4e**) and fused PET/C images (**4f**–**4i**) show no significant interval change in size and metabolic activity of the left axillary lymph node, anterior abdominal wall lesion and omental/mesenteric deposits and lesion near left ischial tuberosity (arrows), suggestive of stable disease

**Fig. 6.**
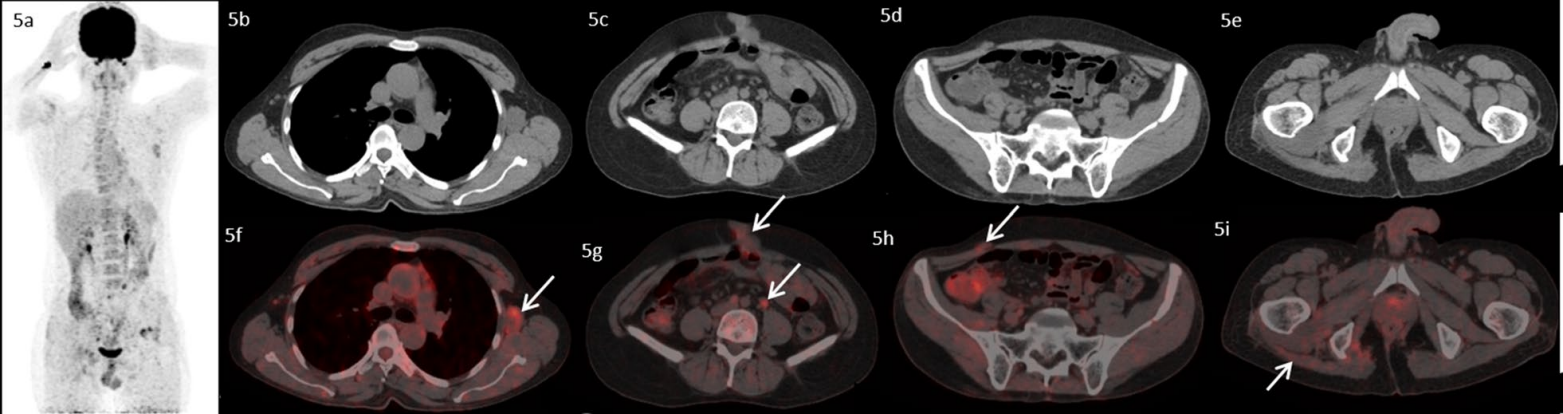
18F-FDG PET/CT images of the patient ten months after initiation of therapy with erlotinib. MIP image (**5a**) transaxial CT images (**5b**–**5e**) and fused PET/C images (**4f**–**4i**) show no significant interval change in size of the left axillary lymph node, anterior abdominal wall lesion and omental/mesenteric deposits and lesion near left ischial tuberosity (arrows) with mild reduction in metabolic activity, overall findings suggestive of stable disease

A 48-year-old male, with no co-morbidities presented in July, 2016 with pain in pelvic region radiating to bilateral lower limbs for one year. Imaging with magnetic resonance imaging (MRI) revealed a large lobulated soft tissue mass in the pelvis. A computed tomography (CT) guided biopsy of soft tissue mass showed lobulated architecture composed of tumor cells having clear to bubbly cytoplasm arranged as cords and embedded in abundant extracellular myxoid matrix with mild atypia. Immunohistochemistry (IHC) of tumor showed membranous positivity for Epithelial Membrane Antigen (EMA) and nuclear positivity for S100; suggestive of chordoma. He underwent partial sacrectomy along with gluteus maximus rotational flap reconstructions in September, 2016. On gross inspection, the specimen measured 6.5 × 5.5 × 3.5 cm. There was a solid gelatinous tumor on cut surface of size 5.5 × 4.5 × 3 cm. On histological examination, the tumor was homogenous in appearance and predominately composed of chondromyxoid stroma with embedded tumor cells arranged in cords and focally in nesting pattern. The individual tumor cells showed moderate cytological pleomorphism with small nuclei and moderate to abundant eosinophilic cytoplasm. There were a few tumor cells with abundant vacuolated cytoplasm which are also called as physaliferous cells. On IHC, tumor cells were diffusely positive for S100 protein and EMA. There was nuclear positivity for brachyury (Fig. 1). Following surgery, post-operative radiotherapy was given by three-dimensional conformal radiation therapy (3D-CRT) technique at a dose of 60 Gray (Gy) in 30 fractions over 6 weeks. He was kept under regular follow up. In November 2018, a follow up MRI scan revealed poorly circumscribed 9.9 × 5.3 × 5.2 cm lesion with lobulated outline adjacent to ischium, infiltrating gluteus muscle and extending along the pelvic wall and infiltrating obturator internus muscle. There was another lobulated soft tissue lesion in anterior abdominal wall infiltrating internal oblique and transversus abdominis muscle. He presented to our center in May, 2019. An fluorodeoxyglucose (FDG)-positron emission tomography (PET) scan in May, 2019 revealed an FDG avid enlarged left axillary node measuring 4.7 × 3.3 cm, multiple mesenteric and peritoneal deposits with largest mesenteric deposit measuring 5.3 × 5.0 cm, FDG avid 6.6 × 6.0 cm soft tissue deposits noted in anterior abdominal wall and 2.7 × 4.7 cm deposit in right external/internal oblique muscle (Fig. 2). The patient received palliative radiotherapy to hemipelvis at a dose of 20 Gy in five fractions over five days. He was started on imatinib at a dose of 400 mg per day from May, 2019. After a brief period of clinical response, he started to have increased local pain in August, 2019. A repeat FDG PET scan done at this time was consistent with disease progression (Fig. 3). In August 2019, patient was started on erlotinib at a dose of 150 mg per day. He showed good response with improvement in general condition and reduction in pain. He developed grade 1 trichomegaly and grade 1 diarrhea on erlotinib. An FDG PET Scan done on October, 2019 showed significant reduction in disease burden consistent with a partial response as per RECIST 1.1 (Fig. 4). Subsequent response assessments at six and twelve (Figs. 5 and 6) months from start of erlotinib therapy revealed stable disease. Immunochemistry on tumor specimen was positive for EGFR expression (Fig. 1). EGFR (exon 18–21) sequencing by real time polymerase chain reaction showed no mutation. MET gene amplification was positive by Fluorescence in Situ Hybridization. Currently the patient is on regular follow-up, and is tolerating therapy well.

## Discussion and conclusions

Surgery remains the backbone of management in sacral chordomas. Although the achievement of wide local resection with negative margins is the goal, the same is usually not always possible because of anatomical complexities of areas where they often arise. In such a setting, adjuvant radiotherapy can be effective in delaying recurrences. A study demonstrated a very good disease control with adjuvant high-dose radiotherapy in primary tumors after resection, but comparatively the outcomes remained poor in the setting of recurrent disease. Park et al. showed excellent disease control with localized radiation post en-bloc resection reporting a 5-year and 10-year survival of 93% and 91% respectively [13].

Although chordoma is a slow-growing disease with low metastatic potential, as many as 30% of patients develop recurrent/advanced disease [14]. In case of recurrent or advanced disease, imatinib is used as a first line agent. The biological rationale of using imatinib comes from its inhibition of PDGFRβ which is expressed on chordomas. This was tested by Stacchiotti et al. using imatinib at a dose of 800 mg per day in patients with advanced PDGFB and/or PDGFRB chordoma, showing a clinical benefit rate of 64% and a median progression free survival (PFS) of 9 months [8].

EGFR, a prototype receptor tyrosine kinase, plays a role in cell proliferation and survival. Weinberger et al. demonstrated strong EGFR and c-MET expression in chordomas and concluded that these may play a role in its growth [15]. In order to elucidate the role of EGFR in pathogenesis of chordoma, Shalaby et al. demonstrated that gain of EGFR copy number is a common event in chordomas and that immunoreactivity for EGFR and p-EGFR is also a frequent finding [9]. They could demonstrate growth inhibitory effect on chordoma cell lines harbouring a EGFR copy number gain by using a reversible tyrosine kinase inhibitor, thus highlighting that aberrant EGFR signalling may be involved in chordoma progression. Using this rationale and the published case reports showing efficacy of erlotinib in chordoma we treated our patient with erlotinib.

Erlotinib has been used as an anti-EGFR agent in chordoma. EGFR inhibitors like lapatinib and erlotinib have also been used; both as monotherapy and in a combination with cetuximab or bevacizumab in advanced/recurrent chordomas, with few patients showing partial responses [10–12, 16–18]. Houessinon et al. reported a patient with clival chordoma who showed a partial response and a sustained benefit for more than 28 months with erlotinib [19]. Our patient showed a partial response within two months of instituting therapy with tolerable side effects—grade 1 trichomegaly and diarrhoea.

In conclusion, erlotinib is a good option in advanced sacral chordoma cases who have progressed on imatinib. As there are fewer than ten cases so far reported in literature showing the efficacy of erlotinib in setting of advanced sacral chordoma that had progressed on imatinib, this case adds to the sparse data on antitumor activity of EGFR inhibition in advanced chordoma, in agreement with other previously reported clinical cases. The targeting of EGFR represents an attractive option in the limited therapeutic armamentarium against advanced chordomas.

## Data Availability

Not applicable.
